# Interleukin-1β (C-511T) Genetic Variant and Major Depressive Disorder: A Systematic Review

**DOI:** 10.3390/ijms27135974

**Published:** 2026-07-03

**Authors:** Bruna Rodrigues Gontijo, Caroline Ferreira Fratelli, Larissa Sousa Silva Bonasser, Calliandra Maria de Souza Silva, Izabel Cristina Rodrigues da Silva

**Affiliations:** 1 Postgraduate Program in Health Sciences and Technologies, Faculty of Ceilândia, University of Brasília (UnB), Brasília-Federal District (DF), Brasília 72220-900, Brazil; brunargontijo.unb@gmail.com (B.R.G.); carolfratelli@gmail.com (C.F.F.); cdssilva@gmail.com (C.M.d.S.S.); 2 Postgraduate Program in Health Sciences, University of Brasília (UnB), Brasília-Federal District (DF), Brasília 70910-900, Brazil; laribonasser@gmail.com; 3 Academic Unit of Biotechnology Engineering (UAEB), Center for Sustainable Development of the Semi-Arid Region (CDSA), Sumé Campus, Federal University of Campina Grande, Sumé 58540-000, Brazil; 4 Clinical Analysis Laboratory, Molecular Pathology Sector, Pharmacy Department, Faculty of Ceilândia, University of Brasília (UnB), Brasília-Federal District (DF), Brasília 72220-900, Brazil

**Keywords:** major depressive disorder (MDD), interleukin-1β (IL1β), C-511T polymorphism (rs16944), genetic variant, cytokines and inflammation, neurodegeneration, antidepressant response, biomarkers, personalized medicine, Psychiatric genetics

## Abstract

Major Depressive Disorder (MDD) has a multifactorial etiology and pathogenesis that significantly impacts an individual’s quality of life. One of the possible correlations for its onset is the activation of inflammatory responses resulting in neurodegeneration and, consequently, the emergence of depressive symptoms. Interleukin-1β is a regulatory cytokine of the immune and nervous systems that acts on several processes, including mood regulation. This systematic review analyzed the *IL1B* (C-511T) (rs16944) variant’s CC and CT genotype frequencies and their associations with MDD in different populations while also verifying the TT genotype’s influence on response to antidepressant therapy. This review involved searching five databases, and articles were selected according to the PECOS inclusion criteria, resulting in eight articles. The findings highlight distinct clinical outcomes: the CC genotype was more frequently associated with greater MDD symptom severity, whereas the TT genotype was predominantly associated with antidepressant treatment response; thus, these associations should not be considered equivalent in terms of susceptibility to disease onset. However, despite these findings, other studies have found no significant association between this genetic variant and MDD. Therefore, further studies across different populations are needed to better understand the role of this polymorphism in the etiology of this disorder.

## 1. Introduction

Major Depressive Disorder (MDD) is characterized by persistent depressed mood, anhedonia, recurrent thoughts of death, and physical and cognitive symptoms, which interfere with the individual’s quality of life [1,2,3]. According to data from the World Health Organization, this condition is the primary contributor to the global burden of disability. Currently, an estimated 322 million people are affected by depression worldwide. Approximately half of these cases are concentrated in Southeast Asia and the Western Pacific, reflecting the fact that these regions host large populations, such as India and China [4].

From 2010 to 2018, the number of adults in the United States with MDD increased by 12.9%, rising from 15.5 million to 17.5 million cases. In the same period, the percentage of adults aged 18 to 34 diagnosed with this condition increased from 34.6% to 47.5%. The additional economic burden associated with MDD also saw a significant jump of 37.9%, rising from US$236.6 billion to US$326.2 billion [5]. Epidemiologically, it is almost twice as prevalent in females as in males, due to both biological and psychological sex differences, as well as environmental factors that act at both the individual and societal levels [6]. Consequently, this difference between the sexes is explained by biological sex-related differences involving hormonal factors, neurostructural particularities, immune system characteristics, inflammatory processes, and metabolic aspects [7].

In this scenario of increasing prevalence and socioeconomic impact of MDD, large-scale stressors have proven capable of further intensifying the disease burden. The COVID-19 pandemic and associated containment measures significantly affected global mental health, leading to a 27.6% rise in MDD prevalence in 2020 alone [8]. This escalation is particularly concerning given the well-established association between MDD and suicidal behavior, as this disorder ranks among the primary psychiatric risk factors for suicide [9,10].

MDD etiology is widely recognized as multifactorial, involving a complex interplay of social, psychological, biological, and genetic factors [3,11]. Genetic polymorphisms have been correlated with MDD, enabling the identification of specific genes that may serve as targets for preventive and therapeutic interventions [12]. Beyond genetic influences, a prominent hypothesis of MDD pathogenesis involves neuroinflammation. This theory suggests that elevated levels of pro-inflammatory cytokines in the central nervous system (CNS), particularly in individuals experiencing neurodegeneration and depressive symptoms, may contribute to the development of the disorder [13].

Interleukin-1β (IL1β) acts as a regulator of the immune and nervous systems, activating B and T lymphocytes and increasing vascular permeability, chemotaxis, and phagocytosis by inflammatory cells [14]. Its *IL1B* gene is located at locus 2q14.1 on chromosome 2. Among the polymorphisms present in the *IL1B* gene, −511C/T (rs16944) is most prominent. This genetic variant has been associated with the modulation of IL-1β production, such that the −511T allele is associated with higher levels of the cytokine [15].

In recent years, the consolidation of Genome-Wide Association Studies (GWAS) has evidenced that MDD presents a highly polygenic architecture, characterized by multiple small-effect variants distributed throughout the genome [16]. Compared to other psychiatric disorders, such as schizophrenia, this disorder presents a lower proportion of identifiable risk variants, whose genotypic effects are generally modest [17].

Consequently, detecting statistically significant associations requires exceptionally large sample sizes, along with consideration of gene–environment interactions and possible epistatic effects [18]. Thus, genetic variants associated with inflammatory pathways may exert context-dependent effects, influencing specific patient subgroups [19].

In this context, the evidence synthesized in the review by Mikhalitskaya et al. [20] highlights the −511C/T (rs16944) polymorphism of the *IL1B* gene as one of the most frequently investigated inflammatory variants in MDD. However, the reported results remain heterogeneous and inconsistent across different populations and study designs, reinforcing that *IL1B*’s genetic contribution must be interpreted in light of the polygenic architecture and biological heterogeneity that characterize this disorder.

A meta-analysis conducted by Ellul et al. [21] evaluated the role of IL1β in MDD, including 53 selected studies from over 1900 identified records. The results demonstrated that elevated blood levels of this cytokine are associated with this psychopathology. Conversely, no consistent associations were found between *IL1B* gene polymorphisms and the risk for the disorder, nor was there a significant effect of antidepressant use on the levels of this cytokine.

In this landscape, marked by methodological advances, inconsistent results, and increasing genetic complexity, a critical synthesis of the available evidence on the *IL1B* −511C/T (rs16944) genetic variant and its relationship with Major Depressive Disorder is warranted. Thus, this systematic review evaluated the influence of this genetic variant on MDD by analyzing CC and CT genotypic frequencies and their potential associations across populations, aiming to clarify potential risk associations and inform diagnostic and therapeutic strategies.

## 2. Methods 

### 2.1. Research Strategy and Selection Criteria

This systematic review was conducted according to the PRISMA guidelines for systematic reviews and meta-analyses and is duly registered with the International Prospective Register of Systematic Reviews (PROSPERO) under CRD420251009366.

The inclusion criteria were based on the following PECOS framework: (1) Population: research participants with Major Depressive Disorder (MDD); (2) Exposure: *IL1B* (C-511T) gene variant (rs16944); (3) Comparison: the *IL1B* (C-511T) genetic variant’s CC/CT genotypic and C allele frequencies; (4) Outcome: the *IL1B* (C-511T) genetic variant’s CC/CT frequency fluctuation in different populations; (5) Study Type: observational and intervention.

The bibliographic search was conducted between March and November 2025 using the following databases: National Library of Medicine (MEDLINE/PubMed), Web of Science, Virtual Health Library (VHL/BVS), Embase, and the Cochrane Library. Original observational or interventional studies in English or Portuguese that investigated the genotypic and allelic effects of the *IL1B* (C-511T) (rs16944) variant on Major Depressive Disorder in humans and included adequate statistical analyses were considered. Studies with incomplete data, systematic reviews, meta-analyses, and conference abstracts were excluded.

The indexed terms were based on the Medical Subject Headings (MESH) vocabulary and the ALLele FREquency Database (ALFRED), which the Yale Center produced for Medical Informatics and catalogs the allele frequencies of different polymorphisms in the human population, free of charge. The descriptors used were ((Depressive Disorder, Major) OR “Major Depressive Disorders” OR “Major Depressive Disorder” OR “Major Depressive”) AND ((Polymorphism, Genetic) OR “Gene Polymorphism” OR “Gene Polymorphisms” OR “Genetic Polymorphisms” OR “Genetic Polymorphism” OR variant OR “Genetic variants” OR variation OR variants) AND ((interleukin 1beta) OR “Interleukin 1beta” OR Catabolin OR “IL-1 beta” OR “Interleukin-1 beta” OR “Interleukin 1 beta” OR IL1B OR IL1β OR “Interleukin-1β” OR “IL-1β”).

### 2.2. Study Selection and Data Extraction

Two reviewers (BR and CS) selected the articles in two stages. The first stage consists of an independent analysis of the study titles and abstracts to determine their eligibility according to the PECOS strategy.

For this initial screening, the Rayyan tool, developed by the Qatar Computing Research Institute (QCRI), was used. This tool also helped remove duplicates. In the second stage, the reviewers (BR and CS) independently evaluated the selected articles’ full text, following the defined eligibility criteria. If any disagreement arose during the evaluation, the third reviewer (IS) was consulted.

Data from the selected studies included information such as author, title, objective, year of publication, country of origin, sample size, main results, *p*-values, and *IL1B* −511C/T polymorphism genotypic frequencies.

### 2.3. Bias Risk in Each Study

Each study’s bias risk was assessed using the Genetic Risk Prediction Studies (GRIPS) guideline, which comprises 25 items designed to promote the selection of high-quality studies and facilitate the comparison and application of data from studies with different designs, methods, or analyses [22]. Two reviewers (BR and CS) independently assessed the presence or absence of these items to assess the methodological quality, results, and discussion of the included articles. In cases of disagreement, a third reviewer (IS) was contacted. Studies that presented 75% or more of the items were considered “good quality.”

## 3. Results

**Figure 1:**
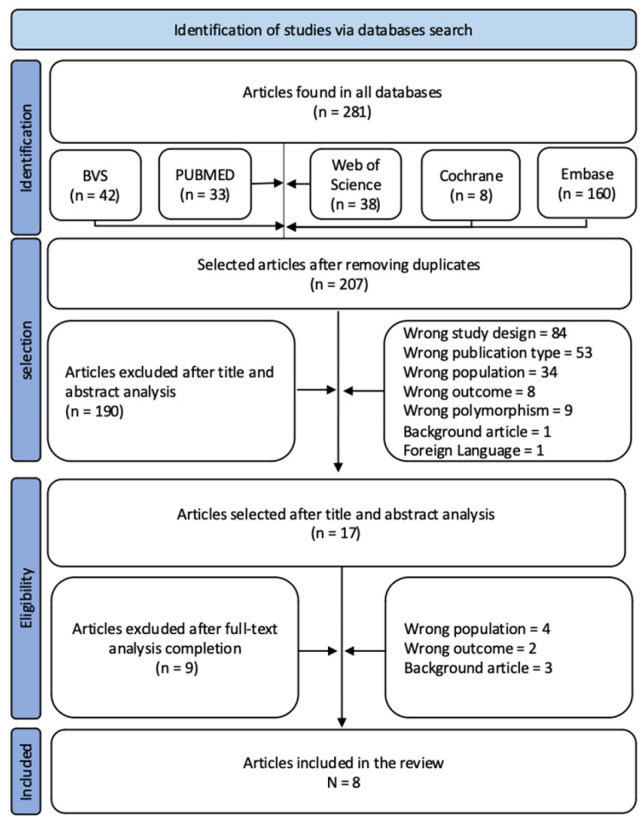
PRISMA bibliographic research flowchart.

From the main research findings, 281 scientific articles were initially identified. After removing duplicates, 207 articles remained. After analyzing the titles and abstracts, 17 articles were selected for further reading. Finally, applying the previously defined inclusion and exclusion criteria based on the PECOS strategy, only eight articles were considered eligible for this systematic review (Figure 1).

Studies excluded during the title and abstract screening stage primarily focused on other psychiatric disorders, non-human populations, narrative or systematic reviews, or experimental designs, or they did not specifically investigate the *IL1B* −511C/T (rs16944) polymorphism in patients with major depressive disorder (MDD), as defined by the PECOS criteria.

Table 1 presents operational definitions for each exclusion category applied during screening to enhance transparency in the selection process. This delineation allows for clear differentiation between exclusions based on outcome, study design, publication type, evaluated population, investigated polymorphisms, theoretical contextualization articles, and language restrictions. The studies excluded after full-text evaluation, along with their respective reasons, are detailed in the Supplementary Materials (Table S2).

Regarding the geographical distribution of the selected articles, 50% originated in Asia, 37% in Europe, and 13% in North America. The eight studies included in this systematic review varied considerably in their designs, populations, and outcomes evaluated, as shown in Table 2. Regarding the *IL1B* C-511T (rs16944) polymorphism association with MDD, some studies demonstrated significant associations with time to antidepressant response [23], age at onset of depression [24], non-remission to treatment [25], and increased risk for MDD [12]. On the other hand, other studies found no statistically significant association between polymorphisms and response to antidepressant treatment, including those by Maciukiewicz et al. [26] and Younger et al. [27]. The polymorphism’s genotypic frequencies varied across the studies, with the C/C and C/T genotypes being the most common.

The studies included in this review varied in their diagnostic criteria, clinical assessment instruments, and genotyping techniques, as shown in Table 3. Most investigations used diagnostic criteria based on the DSM-IV or DSM-IV-TR, whereas only one study used DSM-5 criteria [12]. Depression severity was predominantly assessed using the Hamilton Depression Rating Scale (HAM-D), with differences in the number of items and the assessment design, including both cross-sectional and longitudinal follow-up studies. One study employed the Montgomery–Åsberg Depression Rating Scale (MADRS) as the primary clinical assessment tool [26]. Regarding laboratory aspects, most studies used peripheral blood samples as the source of DNA. However, different genotyping methodologies were employed, including PCR–RFLP and higher-resolution platforms such as MassARRAY, TaqMan OpenArray, and SNaPshot Multiplex.

## 4. Discussion

### 4.1. Inflammation, HPA Axis Dysregulation, and Neuroimmune Mechanisms in Major Depressive Disorder

MDD is widely recognized as a polygenic and multifactorial condition resulting from diverse interactions between genetic, environmental, psychological, and, concomitantly, biological components, with a particular emphasis on the involvement of inflammatory pathways [1,30]. In this context, the effects of individual genetic variants must be interpreted in an integrated manner, considering broader neurobiological and immunological networks that modulate disease susceptibility. Among these mechanisms, interleukin-1β stands out due to its central role in neuroimmune communication, influencing multiple biological pathways directly involved in the pathophysiology of depression [31].

From this perspective, depression is no longer understood merely as a disorder restricted to the central nervous system but is conceived as a systemic condition involving persistent activation of the immune system, alterations in the functioning of the hypothalamus–pituitary–adrenal (HPA) axis, and neurotoxicity processes mediated by inflammatory cytokines [32]. This approach contributes to understanding the high coexistence of this psychopathology with cardiovascular, metabolic, and autoimmune diseases—conditions that share chronic inflammation as a standard feature [7].

Stress plays a central role in the persistent activation of the HPA axis, being responsible for relevant cognitive and emotional alterations. Hyperactivity of this axis represents one of the primary neuroendocrine dysfunctions observed in depression and is associated with increased synthesis and release of corticotropin-releasing hormone (CRH), which stimulates the secretion of adrenocorticotropic hormone (ACTH) by the anterior pituitary, culminating in the release of glucocorticoids by the adrenal cortex [33].

Increased cortisol levels exert negative feedback on the HPA axis through receptors in the hippocampus and hypothalamus, contributing to homeostatic regulation. However, dysfunction of this mechanism favors hypercortisolemia, which induces the activation of indoleamine 2,3-dioxygenase (IDO) in different tissues and immune system cells. Interleukins and interferons potentiate this pathway, redirecting tryptophan metabolism toward the kynurenine pathway, resulting in reduced serotonin synthesis and increased metabolites associated with oxidative stress, excitotoxicity, and the intensification of the inflammatory response [34]. Furthermore, this factor also compromises levels of brain-derived neurotrophic factor (BDNF), impairing neuroplasticity and neurogenesis in MDD [35].

In this context, microglial activation emerges as a central link between systemic inflammation and the neuroendocrine alterations observed in this psychopathology. In addition to acting as the immunocompetent cells of the central nervous system, microglia release pro-inflammatory cytokines such as IL-1β, IL-6, and TNF-α, which can modulate HPA axis activity [7]. In a study by Min et al. [36], patients with MDD showed significantly elevated levels of pro-inflammatory cytokines, particularly TNF-α and IL-6, compared with healthy controls, indicating persistent immunoinflammatory activation.

Beyond the secretion of pro-inflammatory cytokines, microglial activation is associated with alterations in synaptic plasticity, a fundamental process of neuronal communication. The dysregulation of these mechanisms, mediated by microglia through glutamatergic signaling, can compromise neural circuits involved in mood and cognitive regulation, contributing to the pathophysiology of the disorder [37].

Moreover, stress can also activate the inflammasome. This intracellular complex enables the activation of IL-1β from its precursor form, acting as an initial trigger of the inflammatory response at central and peripheral levels. Among the different types described, the NLRP3-mediated inflammasome is the most studied and plays a central role in regulating immunoinflammatory processes. Its activation is influenced by factors, such as reactive oxygen species, which promote caspase-1 activation and the subsequent conversion of pro-IL-1β and pro-IL-18 into active inflammatory cytokines; these species can be generated, among other mechanisms, by activation of the P2X7 purinergic receptor [21,38].

Following its activation, IL-1β acts through receptors expressed in immune cells and the central nervous system and is regulated by endogenous antagonists and anti-inflammatory cytokines, such as IL-4 and IL-10. This signaling activates the nuclear factor-κB (NF-κB) pathway, inducing the expression of inflammatory genes and contributing to the maintenance of the neuroinflammatory response [39].

Regarding genetics, bidirectional Mendelian randomization analyses based on genome-wide association study (GWAS) data indicate a causal relationship between systemic inflammatory regulators and the risk of mental disorders. Thus, inflammation does not constitute a secondary phenomenon but rather an active element in the biological mechanisms underlying the development of these psychopathologies, mediated by genetic factors that modulate the expression and activity of inflammatory cytokines [40].

Polymorphisms in cytokine genes, including *IL1B*, are frequently associated with alterations in circulating levels of these molecules. Such genetic variants appear to directly influence the gene expression of pro-inflammatory cytokines, resulting in a dysregulated inflammatory profile already identified in patients with various psychiatric disorders [20].

Although several biomarkers have been associated with MDD, such as BDNF, cortisol, and inflammatory cytokines, the complexity of the disorder prevents any single marker from being sufficient. Thus, integrated approaches, including genetic and epigenetic factors, have been proposed as promising strategies for better understanding and identifying biomarkers for this disorder [41].

### 4.2. IL1B −511C/T (rs16944) Variant and Its Genotypic Frequency in Major Depressive Disorder

MDD involves biological alterations in which inflammatory signaling pathways exert a relevant modulatory role. From this perspective, cytokines emerge as central mediators of communication between the immune and central nervous systems. Once produced in the periphery, these molecules can reach the brain via specific transport systems, thereby activating neuroinflammatory processes. Concurrently, glial cell activation can sustain cytokine production through feedback loops, interfering with the regulation of monoaminergic neurotransmitter systems and reinforcing the dysfunctional neuroimmune signaling associated with depressive symptomatology [27].

Interleukin-1 beta (IL1B), an important inflammatory mediator, is highly expressed in the brain, especially in the hippocampus, and is strongly involved in memory processes and mood regulation. In the central nervous system (CNS), it is associated with several biological effects, including neuronal proliferation and differentiation, apoptosis induction, and modulation of long-term potentiation [31].

Elevated IL-1 protein levels, especially IL-1β, significantly increase the intensity of inflammation, influencing MDD diagnosis, symptoms, and antidepressant response. Furthermore, studying this cytokine’s expression may be relevant to subsequent pharmacogenetic investigations [12]. The *IL1B* gene, located on the long arm of chromosome 2 (2q14), has a biallelic C/T (rs16944) polymorphism at position -511 of its promoter region.

This genetic variant is associated with varying levels of IL-1β production; individuals homozygous for the −511T allele produce higher levels of IL-1β than those homozygous for the −511C allele. In contrast, the homozygous −511C genotype—correlated with lower IL-1β production—has been associated with greater depressive symptom severity, including higher HAM-D scale scores and a higher occurrence of early insomnia, as well as an earlier onset of geriatric depression when compared to T-allele carriers [15].

A possible explanation for these differences lies in the inflammatory profile associated with the −511C/T polymorphism genotypes. Carriers of the −511TT genotype exhibit higher IL-1β levels compared to −511C individuals. Given that part of the therapeutic effect of antidepressants involves reducing the production of pro-inflammatory cytokines such as IL-1β, it is plausible that individuals with higher basal levels of this cytokine show a greater response to antidepressant treatment, contributing to the differences observed between genotypes [23].

Similarly, the study by Hwang et al. [24] shows that individuals with the homozygous TT genotype produce significantly higher IL-1β levels than heterozygotes (CT), who, in turn, have higher levels than CC homozygotes, demonstrating that patients with higher IL-1β production have less severe depressive symptoms [27].

Consistent with these findings, a systematic review conducted by Shih-Jen Tsai [31] demonstrated that the *IL1B* −511C/T polymorphism is also associated with functional differences in brain circuits involved in emotional regulation. Carriers of the C allele showed lower responsiveness in limbic regions and the anterior cingulate cortex during emotional processing, whereas the TT genotype was associated with more preserved neural patterns, suggesting that higher IL-1β levels may also be associated with less functional impairment and, consequently, less severe depressive symptoms.

Most importantly, the relationship between the −511C/T (rs16944) polymorphism, IL-1β levels, and MDD severity is non-linear. Its influence on IL-1β expression appears to depend on the regulatory context, especially the interaction with the −31T/C (rs1143627) functional polymorphism, with which it exhibits strong linkage disequilibrium. Nevertheless, findings remain inconsistent, with some studies associating the T allele with greater functional activity, while others do not [12,42].

On the other hand, Mikhalitskaya et al. [20] conducted a bibliographic review of scientific databases for studies analyzing cytokine-related genes and investigating genetic overlap among them or cytokine-related genes involved in mental disorders such as major depressive disorder, bipolar disorder, and schizophrenia. According to the analysis, the *IL1B* gene is one of the most investigated cytokine-coding genes, with its involvement in MDD pathophysiology being widely recognized. Among the polymorphisms evaluated, −511C/T (rs16944) stands out, whose C allele has been associated with increased *IL1B* gene expression, leading to elevated IL-1β levels. This polymorphism was also significantly related to the risk of depression, with the CC genotype identified as the highest-risk variant (*p* = 0.001; OR = 1.9; CI 1.3–2.7) in a Russian study [43].

Similarly, in the case–control study conducted by Borkowska et al. [29], no statistically significant association was observed between the −511C/T polymorphism and recurrent depressive disorder, either in the allelic (*p* = 0.2524) or genotypic (*p* = 0.3985) analyses, when comparing the control and patient groups. However, the authors identified a significant association between the C/C (rs16944) genotype and Recurrent Major Depression, while the heterozygous combination at both polymorphic sites is linked by the controls (*p* = 0.064).

Toma et al.’s [12] study also found a statistically significant association between the IL-1β rs16944 polymorphism and the pathology. In the dominant genetic model, individuals with the combined TC + CC genotype exhibited a 2.06-fold increased risk of developing MDD compared to those with the TT genotype. This association was statistically significant (dominant model—TC + CC vs. TT: OR = 2.06; 95% CI: 1.06–3.99; *p* = 0.032).

However, not all ethnically diverse MDD population studies obtained the same results. In Younger et al.’s [27] Taiwanese study, the case and control groups showed no significant difference in genotype (*p* = 0.240) or allele (*p* = 0.134) frequency for the *IL1B* (rs16944) genetic variation. Similarly, in a study with a depressed older adult population [24], no association was found between *IL1B* −511C/T genotypes and susceptibility to geriatric depression (*p* = 0.213); however, depressed individuals with the TT genotype developed depression significantly later, with a difference in onset of 7 years (*p* = 0.021).

Mei-Hung Chi et al. [28] used the HapMap database to compare the allele frequencies of single-nucleotide polymorphisms (SNPs) in several genes, including *IL1B* (rs16944), between Taiwanese patients and four distinct HapMap populations. The researchers identified a significant difference in IL1B SNP allele frequencies only between the Taiwanese control group (TWN-C) and the Yoruba population of Ibadan (YRI) (*p* = 0.048), with the YRI group showing the most significant genetic difference compared to TWN-C. Based on these findings, the authors suggest that geographically closer populations tend to show greater similarity in response to antidepressants.

The discrepancies among the included studies are not solely explained by ethnic differences but also reflect relevant methodological heterogeneities. Diagnostic criteria varied between DSM-IV/DSM-IV-TR and DSM-5, potentially leading to the inclusion of distinct clinical phenotypes. Furthermore, the severity of depression was measured using different instruments and designs—primarily the HAM-D, with variations in the number of items and in its cross-sectional or longitudinal use, as well as the MADRS in clinical trials—limiting the direct comparability of outcomes. In contrast, the DNA source did not emerge as a determining factor, as, when reported, it was predominantly peripheral blood, without a consistent pattern associated with the conflicting results.

In summary, the available findings suggest that ethnic and geographical factors, in isolation, are insufficient to explain the conflicting associations observed for the *IL1B* −511C/T polymorphism. The evidence points to the need to integrate methodological differences across studies—including diagnostic criteria, clinical assessment instruments, and study design—into the understanding of the regulatory mechanisms governing *IL1B* gene expression, thereby allowing for a more consistent and biologically plausible interpretation of the reported results.

### 4.3. IL-1β −511C/T Variant (rs16944) and Pharmacotherapy

André Tadić et al. [23] evaluated the association between genetic variants, specifically *IL1B* C-511T and the *interleukin-1 receptor antagonist (IL-1Ra)*, and antidepressant response to paroxetine and mirtazapine. The study included 101 MDD patients enrolled in a randomized, double-blind, controlled clinical trial. Among patients treated with paroxetine, those homozygous for the *IL1B*-511T allele showed a more favorable treatment response than those with the CT or CC genotype. A significant Time × Genotype interaction was observed using a mixed-effects model for repeated measures (MMRM) (*p* = 0.017). Additionally, mean scores on the Hamilton Rating Scale for Depression (HAMD-17) were lower in TT genotype patients than in CT/CC carriers from day 14 onward, with statistically significant differences observed on day 21 (*p* = 0.028) and day 28 (*p* = 0.025).

Bernhard et al. [25] investigated the relationship between genetic variations in the *IL1B* gene, particularly the variant rs16944, and amygdala and anterior cingulate cortex (ACC) activity in response to emotional stimuli, as well as their impact on response to antidepressant treatment. To identify these variations, the researchers employed the iPLEX multiplex genotyping technique in conjunction with the MassARRAY platform. The study analyzed 256 Caucasian MDD patients (145 female and 111 male). Response to antidepressant treatment over 6 weeks was defined as a reduction in the Rating Scale score. In a subsample of 32 patients, brain activity was assessed using functional magnetic resonance imaging (fMRI) at 3 Tesla during visual presentation of emotional facial expressions. The results revealed a significant association between the CC (rs16944) genotype and non-remission after six weeks of treatment (*p* = 0.034). Furthermore, the number of T alleles positively correlated with increased activity in the subgenual ACC (*p* = 0.00007).

In contrast, Malgorzata Maciukiewicz et al. [26] analyzed polymorphisms in five inflammation-related genes, including *IL1B* C-511T, in relation to treatment response to duloxetine and placebo in patients with severe depression. The study included MDD patients treated with duloxetine (n = 215) or placebo (n = 235) for up to 8 weeks. No significant association was found between the *IL1B* C-511T polymorphism and treatment response to either duloxetine or placebo (*p* = 0.367).

The included studies suggest that the *IL1B* −511C/T (rs16944) polymorphism is associated with distinct outcomes, depending on the context analyzed. While the CC genotype has been more frequently related to increased symptom severity or poorer clinical outcomes in Major Depressive Disorder, the TT genotype appears predominantly associated with antidepressant treatment response in pharmacogenetic studies. This distinction is fundamental to avoiding misleading interpretations of the relationship between the disorder’s clinical characteristics and therapeutic response. Table 4 provides a summary of the effects of the IL1B −511C/T polymorphism.

### 4.4. Selected Articles’ Quality Assessment and Limitations

To perform the quality analysis of the selected studies, 20 out of the 25 criteria of the Genetic Risk Prediction Studies (GRIPS) guideline were applied. These 20 items enabled a structured evaluation of each article’s methodology, results, and discussion. Of the eight selected articles, only one had fewer than 15 of the 20 items evaluated (adequacy <75%) [28]. In contrast, only two articles fulfilled all 20 GRIPS criteria [25,26]. Furthermore, only three articles reported any form of risk model validation [12,25,26], and likewise, only three specified the procedures and data used for model validation [25,26,29]. The results of the GRIPS analysis are presented in Table S1.

Several of the included studies featured small or moderate sample sizes, which may compromise the statistical power required to detect small-effect genetic associations. This aspect is particularly relevant in candidate gene studies, such as those investigating the *IL1B* −511C/T (rs16944) polymorphism, where underpowered designs may yield inconsistent or non-significant results.

Furthermore, the high proportion of studies that failed to identify statistically significant associations raises concerns regarding the possible influence of small-study effects and publication bias. Given the limited number of eligible studies, it was not possible to conduct a formal assessment of this bias; therefore, the available evidence must be interpreted with caution.

## 5. Conclusions

This systematic review demonstrates that, although multiple studies indicate that individuals with the C allele, especially CC homozygotes, present greater severity of depressive symptoms, other research has found no significant association between this genetic variation and MDD. These conflicting results underscore the inconclusive nature of current evidence.

Regarding pharmacogenetics, the *IL1B* C-511T variant has been widely studied for its potential to influence antidepressant response. While some studies report improved treatment outcomes in TT genotype carriers, others found no statistically significant associations, highlighting the necessity for additional research.

This review presents some limitations, including the small number of studies meeting the inclusion criteria, which restricts the representativeness of the evaluated populations and increases susceptibility to small-study effects, especially in candidate gene research. The predominance of studies with non-significant results further suggests the possibility of publication bias, which could not be formally assessed due to the limited number of eligible studies.

Although some statistically significant associations were identified, the effect sizes are modest and do not support the use of the *IL1B* −511C/T polymorphism as a standalone diagnostic biomarker or as a basis for clinical practice changes. Additionally, this variant may serve as a marker SNP in linkage disequilibrium with functionally causal variants in the *IL1B* promoter region, underscoring the need for future studies with larger, more diverse samples and functional analyses.

## Figures and Tables

**Table 1:** Definitions of exclusion criteria applied during the screening process.

Exclusion Category	Operational Definition
Wrong outcome	Studies that evaluated IL-1β levels, inflammatory markers, neurobiological mechanisms, treatment response, pharmacodynamics, safety outcomes, or theoretical constructs without assessing the association between the *IL1B* −511C/T (rs16944) polymorphism and clinical outcomes of Major Depressive Disorder (MDD).
Wrong study design	Studies with methodological designs not aligned with the PECOS protocol, including clinical intervention trials, transdiagnostic analyses, and other non-observational designs.
Wrong publication type	Publications without original human data, such as narrative or systematic reviews, meta-analyses, consensus statements, editorials, theoretical papers, or translational/experimental studies.
Wrong population	Studies conducted in populations not meeting the inclusion criteria, including individuals without a clinical diagnosis of MDD, other psychiatric disorders, transdiagnostic samples, pediatric populations, non-human samples, or patients with non-psychiatric medical conditions.
Wrong polymorphism	Studies that investigated genetic variants other than *IL1B* −511C/T (rs16944) or focused on different inflammatory or pharmacogenetic genes without direct analysis of this specific polymorphism.
Background article	Articles used exclusively for theoretical contextualization that discussed inflammation, IL-1β, or genetic aspects of depression in general, without directly evaluating the association between *IL1B* −511C/T (rs16944) and MDD.
Foreign language	Studies published in languages other than English or Portuguese, with no full-text version available in these languages.

**Table 2:** Comparison of different studies that evaluated the *IL1B* C-511T (rs16944) gene variant’s effect on Major Depressive Disorder (MDD).

Author	Title	Objective	Year	Country	Sample (n)	Results	*p*-Value	Genotypic Frequency
Younger W-Y Yu et al. [27]	Association study of *the interleukin-1 beta* (C-511T) genetic polymorphism with major depressive disorder, associated symptomatology, and antidepressant response.	This study investigated a possible association of the *IL1B* gene’s biallelic (C/T) polymorphism in the promoter region (position -511) with MDD and its clinical symptoms, as well as its therapeutic response.	2003	Taiwan	MDD Group: n = 157 M = 70 F = 49 Control Group: n = 112	No significant differences were found between any of the genotypes and allele frequencies when comparing the case and control groups.	Frequencies: Genotypes (*p* = 0.240) Alleles (*p* = 0.134)	MDD C/C = 31.8% (n = 50) C/T = 53.5% (n = 84) T/T = 14.6% (n = 23) Control C/C = 23.2% (n = 26) C/T = 57.1% (n = 64) T/T = 19.6% (n = 22)
Tadić et al. [23]	Association analysis between variants of the *interleukin-1β* and the *interleukin-1 receptor antagonist* gene and antidepressant treatment response in major depression	To verify the possible effect of two polymorphisms, including the *IL1B* C-511T gene, on the result of the antidepressant response to treatment with the antidepressants paroxetine and mirtazapine in patients with major depression.	2008	Germany	MDD group ^1^ n = 116 M = 27 F = 74	Patients homozygous for the T allele responded more quickly and strongly to paroxetine treatment compared to those with the other alleles. A significant interaction was also observed between time and genotype using a mixed-effects model for repeated measures (MMRM) ^2^. TT carriers showed lower mean scores on the Hamilton Rating Scale for Depression (HAMD-17) compared to CT or CC carriers, starting from day 14, with statistically significant differences on days 21 and 28.	Time × Genotype Interaction (MMRM) (*p* = 0.017) Difference in HAMD-17 scores on day 21 (*p* = 0.028) Difference in HAMD-17 scores on day 28 (*p* = 0.025)	Paroxetine CC = 30.6% (n = 15) CT = 55.1% (n = 27) TT = 14.3% (n = 7) Mirtazapine CC = 38.5% (n = 20) CT = 51.9% (n = 27) TT = 9.6% (n = 5)
Jen-Ping Hwang et al. [24]	*Interleukin-1 beta* −511C/T genetic polymorphism is associated with age of onset of geriatric depression	Determine whether the *IL1B* gene’s biallelic functional −511C/T polymorphism in the promoter region affects vulnerability to geriatric depression and its manifestations, including age of onset, depression severity, and cognitive function.	2009	China	Older adults with MDD: n = 125 M = 76 F = 49 Control Group: n = 282 M = 193 F = 89	There was no significant association between the genotypes of this genetic variant and susceptibility to geriatric depression, depression severity, or cognitive function. The only finding suggests that the *IL1B* −511C/T polymorphism may be related to the later age of onset, being 7 years later.	*IL1B* −511C/T genotypes and susceptibility to geriatric depression (*p* = 0.213) Age of onset of depression across genotypes (*p* = 0.021)	MDD C/C = 32.8% (n = 41) C/T = 45.6% (n = 57) T/T = 21.6% (n = 27) Control C/C = 24.8% (n = 70) C/T = 53.5% (n = 151) T/T = 21.6% (n = 61)
Bernhard T. Baune et al. [25]	The *Interleukin 1 Beta (IL1B)* Gene Is Associated with Failure to Achieve Remission and Impaired Emotion Processing in Major Depression	Investigate the association between *IL1B* genetic variants, including −511C/T, and the responsiveness of the amygdala and anterior cingulate cortex (ACC) to emotional stimuli and the response to antidepressant treatment.	2010	Germany	MDD Group: n = 256 M = 111 F = 145	Although the *IL1B* rs16944 genetic variation’s genotype distribution did not differ significantly (*p* = 0.23), the CC rs16944 genotype was significantly associated with non-remission after 6 weeks of treatment (*p* = 0.02). Imaging analyses also revealed a significant positive association, showing that the C rs16944 allele was linked to reduced responsiveness of the amygdala and anterior cingulate cortex (ACC) to emotional stimuli.	Genotypes (*p* = 0.23) Significant associations of the CC rs16944 genotype with non-remission after 6 weeks of antidepressant treatment (*p* = 0.034).	MDD: TT: 25.8% (n = 66) TC: 45.7% (n = 117) CC: 28.5% (n = 73) ^2^
Mei-Hung Chi et al. [28]	Comparison of Antidepressant Efficacy-related SNPs Among Taiwanese and Four Populations in the HapMap Database	The allele frequencies of related SNPs, including *IL1B* −511C/T, to antidepressant efficacy were compared between the Taiwanese population and other populations in the HapMap database.	2011	Taiwan	MDD Group: n = 198 Control Group: n = 106 M = 35 F = 71	No significant differences were observed between Taiwanese patients (TWN-P) and Taiwanese controls (TWN-C). However, significant differences emerged when comparing TWN-C to the other four ethnic groups: (1) Han Chinese in Beijing (CHB), (2) individuals of European ancestry residing in the USA (CEU), (3) Japanese in Tokyo (JPT), and (4) Yoruba in Ibadan (YRI). Notably, a statistically significant difference in the *IL1B* gene SNPs’ allele frequency was found only between TWN-C and YRI (*p* = 0.048).	TWN-P vs. TWN-C (*p* = 0.886) TWN-C vs. JPT (*p* = 0.887) TWN-C vs. CHB (*p* = 0.478) TWN-C vs. CEU (*p* = 0.225) TWN-C vs. YRI (*p* = 0.048)	TWN-P T: 0.420 C: 0.580 TWP-C T: 0.429 C: 0.571 CHB T: 0.477 C: 0.523 CEU T: 0.345 C: 0.655 JPT T: 0.444 C: 0.556 YRI T: 0.566 C: 0.434 ^2^
Paulina Borkowska et al. [29]	*Interleukin-1beta* Promoter (−31T/C and −511C/T) Polymorphisms in Major Recurrent Depression	This study aimed to conduct a case–control study, verifying two *IL1B* polymorphisms (−31T/C and −511C/T) in the promoter region in patients suffering from severe recurrent depression.	2011	Poland	MDD Group: n = 121 F: 94 M: 27 Control Group: n = 206 M = 131 F = 75	A specific haplotype containing the T allele at position -511 showed a trend towards statistical significance in patients compared to controls. Furthermore, correspondence analysis revealed that the C/C genotype at-511 is associated with recurrent major depressive disorder.	Haplotype frequencies in the patient and control groups (*p* = 0.064)	MDD C/C = 44.7% (n = 42) C/T = 49% (n = 52) T/T = 3.3% (n = 3) Control C/C = 36.9% (n = 76) C/T = 58.3% (n = 120) T/T = 4.8% (n = 10)
Malgorzata Maciukiewicz et al. [26]	Genetic variation in *IL-1β*, *IL-2*, *IL-6*, *TSPO* and *BDNF* and response to duloxetine or placebo treatment in major depressive disorder	This study investigated polymorphisms of five inflammation-related genes, including *IL1B* −511C/T, for association with response to duloxetine and placebo in patients with major depression.	2015	Canada	All patients n = 411 F: 275 M: 136 Placebo group n = 215 F: 142 M: 73 Duloxetine n = 196 F: 133 M: 63	No significant associations were observed between this SNP and response to duloxetine or placebo treatment in MDD patients. The minor allele frequency was 36% in the analyzed sample.	Duloxetine 0.367 (ΔMADRS), 0.172 (resp.) Placebo 0.388 (ΔMADRS), 0.577 (resp.)	Genotypic frequency not reported. Minor allele frequency (MAF): 36%.
Faria Mehreen Toma et al. [12]	*Interleukin-1β* rs16944 and rs1143627 polymorphisms and risk of developing major depressive disorder: A case–control study among Bangladeshi population	The authors’ objective is to verify the correlation between *IL1B* rs16944 and rs1143627 polymorphisms and susceptibility to MDD.	2025	Bangladesh	MDD Group: n = 100 F: 73 M: 27 Control Group: n = 70 M = 19 F = 51	The *IL1B* rs16944 polymorphisms revealed that the combined TC + CC genotype in the dominant model showed a 2.06-fold increased risk of developing MDD compared with the common homozygous TT (OR = 2.06).	Dominant model (TC + CC vs. TT) (*p* = 0.032)	MDD C/C = 4% (n = 4) C/T = 40% (n = 40) T/T = 56% (n = 56) Control C/C = 0% (n = 0) C/T = 27.1% (n = 19) T/T = 72.9% (n = 51)

^1^—In the study by Tadić et al. [23], a total of 269 participants were enrolled; however, only 116 provided informed consent for blood sample collection used in DNA genotyping and molecular analysis. Twelve individuals were excluded due to missing baseline data on the 17-item Hamilton Rating Scale for Depression (HAMD-17). Additionally, three patients were excluded from the *IL1β* C-511T gene analysis due to genotyping failure. ^2^—Genotypes initially reported as A/G were converted to the C/T format to ensure consistency across studies, in accordance with the reference allele orientation for rs16944.

**Table 3:** Methodological characteristics of the studies included in the review.

Study	Diagnostic Criteria	Structured Interview	Depression Severity Scale	Longitudinal Assessment	DNA Source	Genotyping Method
Yu et al., 2003 [27]	DSM-IV	No (clinical interview by senior psychiatrist)	HAM-D ^1^ (21 items)	No	Blood	PCR–RFLP
Tadić et al., 2008 [23]	DSM-IV	No (clinical interview by trained raters)	HAM-D (17 items) MADRS used only for suicide risk exclusion	Yes	Blood	PCR–RFLP
Hwang et al., 2009 [24]	DSM-IV	Yes (Mini-International Neuropsychiatric Interview—MINI)	HAM-D (17 items)	No	Not explicitly reported	PCR–RFLP
Baune et al., 2010 [25]	DSM-IV	Yes (Structured clinical interview—SCID-I)	HAM-D (21 items)	Yes	Not explicitly reported	MassARRAY iPLEX^®^
Borkowska et al., 2011 [29]	DSM-IV-TR	Yes (SCID-I)	HAM-D(cross-sectional)	No	Blood	PCR–RFLP
Chi et al., 2011 [28]	DSM-IV	Yes (Chinese version of the Mini International Neuropsychiatric Interview (MINI) for controls)	HAM-D (baseline)	No	Blood	SNaPshot Multiplex + LightCycler
Maciukiewicz et al., 2015 [26]	DSM-IV-TR	Yes (clinical trial setting)	MADRS ^2^	Yes	Blood	TaqMan OpenArray^®^
Toma et al., 2025 [12]	DSM-5	No	HAM-D (baseline)	No	Blood	PCR–RFLP

^1^—Hamilton Depression Rating Scale; ^2^—Montgomery–Åsberg Depression Rating Scale.

**Table 4:** Functional Dichotomy of the *IL1B* −511C/T Polymorphism: Clinical Outcomes vs. Pharmacogenetic Profiles.

Feature	CC Genotype	TT Genotype
Primary Association	Higher Symptom Severity	Improved Treatment Response
IL-1β Levels	Generally Lower	Generally Higher
Clinical Impact	Poorer prognosis/Early onset	Better response to SSRIs

## Data Availability

No new data were created or analyzed in this study. Data sharing is not applicable to this article.

## Supplementary Materials

The following supporting information can be downloaded at: https://www.mdpi.com/article/10.3390/ijms27135974/s1. References [44,45,46,47,48,49,50,51,52] are cited in the Supplementary Materials.
