# Supplementary material for: Interleukin-1β (C-511T) Genetic Variant and Major Depressive Disorder: A Systematic Review
Source: Int J Mol Sci. 2026 Jul 3;27(13):5974. doi: 10.3390/ijms27135974 (PMC13361593; doi:10.3390/ijms27135974)
Supplement: Supplementary file 1 [file ijms-27-05974-s001.zip › ijms-4055564-supplementary.pdf]

Table S1. The systematic review selected articles' quality assessment according to the Genetic Risk Prediction Studies (GRIPS) guideline.

	Section/Topic	Younger W-Y Yu et al. [1]	Tadić et al. [2]	Jen-Ping Hwang et al. [3]	Bernhard T. Baune et al. [4]	Mei-Hung Chi et al. [5]	Paulina Borkowska et al[6]	Malgorzata Maciukiewicz et al. [7]	Faria Mehreen Toma, et al. [8]
	Methods								
**Study desing and Setting**	4) Present key elements of study design early in the paper and Describe the setting, locations, and relevant dates, including periods of recruitment, exposure, follow-up, and data collection	x	x	x	x	x	x	x	x
**Participants**	5) Describe eligibility criteria for participants, and sources and methods of selection of participants	x	x	x	x	x	x	x	x
**Variables: Definition**	6) Clearly define all participant characteristics, risk factors and outcomes. Clearly define genetic variants using a widely-used nomenclature system	x	x	x	x	x	x	x	x
**Variables: Assessment**	7) (a) Describe sources of data and details of methods of assessment (measurement) for each variable.	x	x	x	x	x	x	x	x
	(b) Give a detailed description of genotyping and other laboratory methods.	x	x	x	x	x	x	x	x
	8) (a) Describe how genetic variants were handled in the analyses	x	x	x	x	x	x	x	x

**Variables: Coding**	(b) Explain how other quantitative variables were handled in the analyses. If applicable, describe which groupings were chosen, and why.	x	x	x	x	x	x	x	x
**Analysis: Risk model construction**	9) Specify the procedure and data used for the derivation of the risk model. Specify which candidate variables were initially examined or considered for inclusion in models. Include details of any variable selection procedures and other modelbuilding issues. Specify the horizon of risk prediction (e.g., 5-year risk).				x		x	x	x
**Analysis: Validation**	10) Specify the procedure and data used for the validation of the risk model.				x		x	x	
**Analysis: Missing data**	11) Specify how missing data were handled		x		x		x	x	x
**Analysis: Statistical methods**	12) Specify all measures used for the evaluation of the risk model including, but not limited to, measures of model fit and predictive ability	x			x		x	x	x
**Analysis: Other**	13) Describe all subgroups, interactions, and exploratory analyses that were examined	x	x	x	x	x	x	x	
	Results								
**Participants**	14) Report the numbers of individuals at each stage of the study. Give reasons for nonparticipation at each stage. Report the number of participants not genotyped, and reasons why they were not genotyped.	x	x	x	x	x	x	x	x

**Descriptives: Population**	15) Report demographic and clinical characteristics of the study population, including risk factors used in the risk modeling.	x	x	x	x	x	x	x	x
**Variables: Definition**	16) Report unadjusted associations between the variables in the risk model(s) and the outcome. Report adjusted estimates and their precision from the full risk model(s) for each variable.	x	x	x	x		x	x	x
**Descriptives: Model estimates**	17) Descriptives: Model estimates	x	x	x	x		x	x	x
**Assessment**	18) Report measures of model fit and predictive ability, and any other performance measures, if pertinent.				x		x	x	x
**Validation**	19) Report any validation of the risk model(s)				x			x	x
**Other analyses**	20) Present results of any subgroup, interaction, or exploratory analyses, whenever pertinent	x	x	x	x	x	x	x	x
	**Discussion**								
**Limitations**	21) Discuss limitations and assumptions of the study, particularly those concerning study design, selection of participants, and measurements and analyses, and discuss their impact on the results of the study	x	x	x	x	x	x	x	x
**Interpretation**	22) Give an overall interpretation of results considering objectives, limitations, multiplicity of analyses, results from similar studies, and other relevant evidence	x	x	x	x	x	x	x	x

**Generalizability**	23) Discuss the generalizability and, if pertinent, the health care relevance of the study results	x	x	x	x	x	x	x	x

X – present.

Table S2. Studies excluded after full-text assessment, along with the reasons for exclusion, according to PRISMA criteria.

Authors	Year	Title	Reason for exclusion (full-text assessment)	PRISMA exclusion criterion
**Misener et al. [9]**	2009	*Tagging SNP association study of the IL1B gene and childhood-onset mood disorders*	Investigated mood disorders with childhood or adolescent onset, not corresponding to the adult population with clinically diagnosed Major Depressive Disorder defined in the PECOS criteria.	**Wrong population**
**Ridout et al. [10]**	2014	*Interleukin 1B gene variation and internalizing symptoms in maltreated preschoolers*	Although Major Depressive Disorder and PTSD were considered, the study was conducted in a pediatric population and focused on internalizing symptoms during early childhood, which does not correspond to the adult clinical MDD population defined in the PECOS criteria.	**Wrong population**
**Mueller, D.J [11]**	2015	*Genetic variation of inflammatory markers (IL-1beta, IL-2, IL6, TSPO) and BDNF in response to treatment with duloxetine and placebo*	Available only as a conference abstract and lacking extractable data specific to the IL1B −511C/T (rs16944) polymorphism; therefore, the study was used solely for contextual background and did not meet the inclusion criteria for data extraction.	**Background article**
**Kovacs et al. [12]**	2016	*Effects of IL1B single nucleotide polymorphisms on depressive and anxiety symptoms are determined by severity and type of life stress*	Evaluated depressive and anxiety symptoms in a general population sample and gene–environment interactions, without a primary focus on clinically diagnosed MDD or on the IL1B −511C/T (rs16944) polymorphism as the main outcome.	**Wrong population**
**Cohen-Woods et al. [13]**	2017	*Interaction between childhood maltreatment on immunogenetic risk in depression*	Although rs16944 was included among several inflammatory variants, the study focused on gene–environment interactions and did not report explicit genotype frequencies (CC/CT/TT) or isolate the association of this polymorphism with Major Depressive Disorder outcomes, precluding data extraction according to the PECOS criteria.	**Wrong outcome**

**Marshe V. et al. [14]**	2017	*Investigating associations between IL-1β, IL-2, IL-6, TSPO and BDNF variants and response to duloxetine or placebo treatment in patients with major depression*	Available only as a conference abstract; although inflammatory genes were investigated, no specific or extractable results were reported for the IL1B −511C/T (rs16944) polymorphism. The study was therefore used solely for contextual background and did not meet the inclusion criteria for data extraction.	**Background article**
**Draganov et al. [15]**	2018	*Association study of polymorphisms within inflammatory genes and treatment resistant depression*(conference abstract)	Conference abstract without full methodological details or complete results, precluding adequate assessment of eligibility.	**Background article**
**Draganova et al. [16]**	2019	*Association study of polymorphisms within inflammatory genes and methylation status in treatment response in major depression*	Although the IL1B −511C/T (rs16944) polymorphism was included among several inflammatory variants, the study focused on treatment response and DNA methylation profiles without reporting explicit genotype frequencies (CC/CT/TT) or isolated associations for this variant, precluding data extraction according to the PECOS criteria.	**Wrong outcome**
**McQuaid et al. [17]**	2019	*Understanding the relation between early-life adversity and depression symptoms: The moderating role of sex and an interleukin-1β gene variant*	Included a non-clinical sample and focused on depressive symptoms rather than clinically diagnosed Major Depressive Disorder.	**Wrong population**

Reference

1. Yu, Y.W.Y.; Chen, T.J.; Hong, C.J.; Chen, H.M.; Tsai, S.J. Association Study of the Interieukin-Ibeta (C-5I IT) Genetic Polymorphism with Major Depressive Disorder, Associated Symptomatology, and Antidepressant Response. *Neuropsychopharmacology* 2003, *28*, 1182–1185, doi:10.1038/sj.npp.1300172.

2. Tadić, A.; Rujescu, D.; Müller, M.J.; Kohnen, R.; Stassen, H.H.; Szegedi, A.; Dahmen, N. *Association Analysis between Variants of the Interleukin-1β and the Interleukin-1 Receptor Antagonist Gene and Antidepressant Treatment Response in Major Depression*; 2008; Vol. 4;.

3. Hwang, J.P.; Tsai, S.J.; Hong, C.J.; Yang, C.H.; Hsu, C.D.; Liou, Y.J. Interleukin-1 Beta -511C/T Genetic Polymorphism Is Associated with Age of Onset of Geriatric Depression. *Neuromolecular Med.* 2009, *11*, 322–327, doi:10.1007/s12017-009-8078-x.

4. Baune, B.T.; Dannlowski, U.; Domschke, K.; Janssen, D.G.A.; Jordan, M.A.; Ohrmann, P.; Bauer, J.; Biros, E.; Arolt, V.; Kugel, H.; et al. The Interleukin 1 Beta (IL1B) Gene Is Associated with Failure to Achieve Remission and Impaired Emotion Processing in Major Depression. *Biol. Psychiatry* 2010, *67*, 543–549, doi:10.1016/j.biopsych.2009.11.004.

5. Mei-Hung Chi; Sheng-Yu Lee; Hui-Hua Chang; Yen-Kuang Yang; Eugene Lin; Po-See Chen *Different Racial Antidepressant Response*;

6. Borkowska, P.; Kucia, K.; Rzezniczek, S.; Paul-Samojedny, M.; Kowalczyk, M.; Owczarek, A.; Suchanek, R.; Medrala, T.; Kowalski, J. Interleukin-1beta Promoter (-31T/C and -511C/T) Polymorphisms in Major Recurrent Depression. *Journal of Molecular Neuroscience* 2011, *44*, 12–16, doi:10.1007/s12031-011-9507-5.

7. MacIukiewicz, M.; Marshe, V.S.; Tiwari, A.K.; Fonseka, T.M.; Freeman, N.; Rotzinger, S.; Foster, J.A.; Kennedy, J.L.; Kennedy, S.H.; Müller, D.J. Genetic Variation in IL-1β, IL-2, IL-6, TSPO and BDNF and Response to Duloxetine or Placebo Treatment in Major Depressive Disorder. *Pharmacogenomics* 2015, *16*, 1919–1929, doi:10.2217/pgs.15.136.

8. Toma, F.M.; Kalam, K.T.; Haque, M.A.; Reza, S.; Akter, R.; Islam, M.S.; Islam, M.R.; Nahar, Z. Interleukin-1β Rs16944 and Rs1143627 Polymorphisms and Risk of Developing Major Depressive Disorder: A Case-Control Study among Bangladeshi Population. *PLoS One* 2025, *20*, doi:10.1371/journal.pone.0317665.

9. Misener, V.L.; Gomez, L.; Wigg, K.G.; King, N.; Kiss, E.; Daróczi, G.; Kapornai, K.; Tamás, Z.; Mayer, L.; Gádoros, J.; et al. Tagging SNP Association Study of the IL-1β Gene (IL1B) and Childhood-Onset Mood Disorders. *American Journal of Medical Genetics, Part B: Neuropsychiatric Genetics* 2009, *150*, 653–659, doi:10.1002/ajmg.b.30885.

10. Ridout, K.K.; Parade, S.H.; Seifer, R.; Price, L.H.; Gelernter, J.; Feliz, P.; Tyrka, A.R. Interleukin 1B Gene (IL1B) Variation and Internalizing Symptoms in Maltreated Preschoolers. *Dev. Psychopathol.* 2014, *26*, 1277–1287, doi:10.1017/S0954579414001023.

11. Mueller, D.J. Genetic Variation of Inflammatory Markers (IL-1beta, IL-2, IL6, TSPO) and BDNF in Response to Treatment with Duloxetine and Placebo. *Biol. Psychiatry* 2015, *77*, 224S.

12. Kovacs, D.; Eszlari, N.; Petschner, P.; Pap, D.; Vas, S.; Kovacs, P.; Gonda, X.; Juhasz, G.; Bagdy, G. Effects of IL1B Single Nucleotide Polymorphisms on Depressive and Anxiety Symptoms Are Determined by Severity and Type of Life Stress. *Brain Behav. Immun.* 2016, *56*, 96–104, doi:10.1016/j.bbi.2016.02.012.

13. Cohen-Woods, S.; Fisher, H.L.; Ahmetspahic, D.; Douroudis, K.; Stacey, D.; Hosang, G.M.; Korszun, A.; Owen, M.; Craddock, N.; Arolt, V.; et al. Interaction between Childhood Maltreatment on Immunogenetic Risk in Depression: Discovery and Replication in Clinical Case-Control Samples. *Brain Behav. Immun.* 2018, *67*, 203–210, doi:10.1016/j.bbi.2017.08.023.

14. Marshe, V.; Maciukiewicz, M.; Tiwari, A.K.; Freeman, N.; Kennedy, J.L.; Rotzinger, S.; Kennedy, S.; Müller, D. Investigating associations between IL-1β, IL-2, IL-6, TSPO and BDNF variants and response to duloxetine or placebo treatment in patients with major depression. Eur. Neuropsychopharmacol. 2017, 27, S139–S356 (Abstract Sa77).

15. M. Draganov 1; M.J. Arranz; E. Alvarez; J. De Diego-Adeliño; M. Jubero; D. Puigdemont Association Study of Polymorphisms within In- FLammatory Genes and Treatment Resistant Depression. 2018, *29*, doi:10.1016/j.euroneuro.2018.11.1029.

16. Draganov, M.; Arranz, M.J.; Salazar, J.; de Diego-Adeliño, J.; Gallego-Fabrega, C.; Jubero, M.; Carceller-Sindreu, M.; Portella, M.J. Association Study of Polymorphisms within Inflammatory Genes and Methylation Status in Treatment Response in Major Depression. *European Psychiatry* 2019, *60*, 7–13, doi:10.1016/j.eurpsy.2019.05.003.

17. McQuaid, R.J.; Gabrys, R.L.; McInnis, O.A.; Anisman, H.; Matheson, K. Understanding the Relation between Early-Life Adversity and Depression Symptoms: The Moderating Role of Sex and an Interleukin-1β Gene Variant. *Front. Psychiatry* 2019, *10*, doi:10.3389/fpsyt.2019.00151.
